# DUAL SENSORY LOSS AND COGNITIVE TEST PERFORMANCE IN OLDER ADULTS IN INDIA

**DOI:** 10.1093/geroni/igad104.2827

**Published:** 2023-12-21

**Authors:** Ethan Wang, Jennifer Deal

**Affiliations:** Johns Hopkins University, Baltimore, Maryland, United States; Johns Hopkins University, Baltimore, Maryland, United States

## Abstract

Dementia is rising in the global population, especially in low- and middle-income countries like India. Hearing and vision loss are risk factors for cognitive decline/dementia, but studies have primarily been in high income countries. This study aims to investigate the association between dual sensory loss and cognitive function with specific domains among the older Indian population. Data was from the 2017-2019 Wave 1 of the population-based Longitudinal Aging Study in India. The analysis sample consists of 27,975 individuals aged 60 years and older in 35 states and union territories. Dual sensory loss was determined by respondents’ self-reported, perceived difficulty regarding hearing and vision function. Linear regression was used to model associations of dual sensory loss with global cognitive performance, summarized as a z-score, and in the domains of memory, orientation, arithmetic, and executive function, adjusted for demographics and health characteristics. Overall, 5.9% had hearing loss, 24.1% had vision loss, and 3.4% had both. In fully adjusted models, hearing loss only, vision loss only, and dual sensory loss were associated with a change of -0.08 (95%CI: -0.1, -0.04), -0.11 (95%CI: -0.12, -0.09), and -0.22 (95%CI: -0.27, -0.18) standard deviations, respectively. Associations were also observed for all domains. Poor hearing and/or vision loss is associated with lower cognitive performance across various cognitive domains in older adults in India. The association was strongest for those with dual sensory loss. Rehabilitative interventions for hearing and vision may be an additional resource in reducing dementia risk in India.

